# Quantifying whole lung iron oxide deposition with dual-energy CT for diagnosis of arc-welders’ pneumoconiosis

**DOI:** 10.1007/s00330-025-11839-z

**Published:** 2025-07-25

**Authors:** Weiling Wang, Yuan Ou, Lixin Lu, Minxue Wang, Ming Lu, Suping Chen, Jianyu Li, Ling Luo, Bingru Liu, Qiong Yang

**Affiliations:** 1 Department of Radiology, Guiqian International General Hospital, Guiyang, People’s Republic of China; 2 CT Research Center, GE HealthCare, Changsha, People’s Republic of China

**Keywords:** Pneumoconiosis, Iron, Computed tomographic scintigraphy

## Abstract

**Objectives:**

Accurately quantify pulmonary iron oxide by dual-energy CT (DECT) and evaluate its diagnostic potential in arc-welders’ pneumoconiosis (AWP).

**Materials and methods:**

This prospective, single‑center diagnostic accuracy study (April 2024 to October 2024) included three groups: welders, mimic-imaging, and healthy controls. DECT quantified whole-lung Fe_2_O_3_ density (mg/cm³) [*D*_Fe2O3_] and total Fe_2_O_3_ mass (mg) [Total-Fe_2_O_3_]. Maximal diameter and *D*_Fe2O3_ for the largest nodule and the subjective grading on imaging features were also collected. Receiver operating characteristic (ROC) analysis was used to evaluate diagnostic performance. In vitro experiments, ten 30 mL tubes containing Fe_2_O_3_ solutions (0–12 mg/mL, in triplicate) were scanned immediately after preparation. ROIs were analyzed, and averaged values were linearly regressed with actual concentrations.

**Results:**

In vitro experiments showed a strong correlation between measured and actual Fe_2_O_3_ concentrations (*r* = 1.00, *p* < 0.01). One hundred forty participants were included: 50 welders (mean age, 44.5 years ± 20.25；47 male), 35 mimic-imaging controls (mean age, 51.0 years ± 10.0; 34 male), and 55 healthy controls (mean age, 48.0 years ± 19.0; 41 male). Welders had higher $D_{{{\rm{Fe}}}_2{{\rm{O}}}_3}$ (0.934 ± 0.50 mg/cm³) and Total-Fe_2_O_3_ (4082.6 ± 2503.1 mg) than mimic (0.346 ± 0.28 mg/cm^3^; 1376.1 ± 1514.9 mg) and healthy controls (0.371 ± 0.24 mg/cm^3^; 1374.1 ± 896.2 mg) (all *p* < 0.001). *D*_Fe2O3_ distinguished welders from healthy controls with AUC 0.911 [95% CI: 0.840–0.958], sensitivity 82.0%, specificity 92.7%, and from mimic controls with AUC 0.900 [95% CI: 0.816–0.954], sensitivity 84.0%, specificity 82.9%. In vitro experiments showed a strong correlation (*r* = 1.00, *p* < 0.01) between actual and measured concentrations.

**Conclusion:**

Quantification of pulmonary iron oxide deposition using DECT can aid in the differential diagnosis of AWP.

**Key Points:**

***Question*** *Due to overlapping imaging features between AWP and other diffuse pulmonary diseases, a definitive diagnosis is difficult based solely on imaging*.

***Findings*** *Pulmonary D*_*Fe2O3*_ *and Total-Fe*_*2*_*O*_*3*_ *by DECT showed high diagnostic accuracy (AUCs* *>* *0.90) for distinguishing welders’ pneumoconiosis from both mimic-imaging and healthy controls*.

***Clinical relevance*** *Non-invasive DECT Fe*_2_*O*_*3*_ *quantification improves the differential diagnosis of AWP, and enables targeted monitoring of occupational exposure, enhancing patient outcomes and occupational health management*.

**Graphical Abstract:**

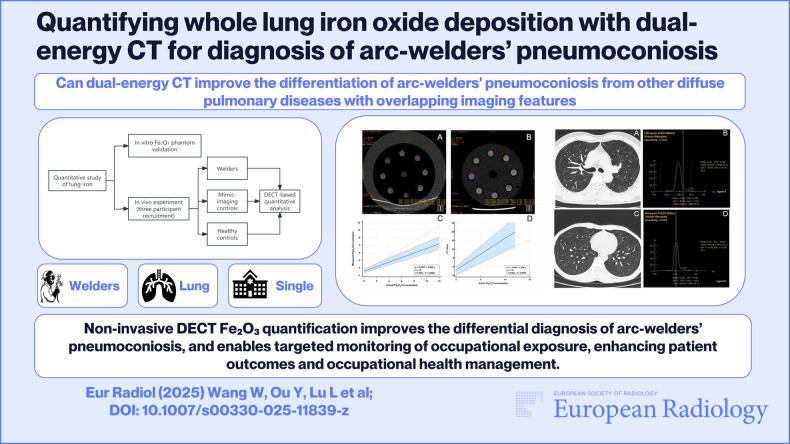

## Introduction

Arc-welders’ pneumoconiosis (AWP) is a pneumoconiosis caused by prolonged inhalation of high concentrations of welding fumes. While some studies suggest that AWP does not lead to pulmonary fibrosis or impaired lung function [1], other research indicates that excessive iron fumes can cause irreversible fibrosis [2]. Prolonged exposure can cause interstitial, perivascular, and peribronchial fibrosis, worsening with high-dose exposure and potentially leading to lung cancer, highlighting the importance of early diagnosis [2].

Currently, diagnosing AWP relies on a comprehensive approach that includes clinical evaluation (occupational history), imaging, and pathological assessment. CT imaging stands out as a significant diagnostic tool, commonly featuring centrilobular nodules/ground-glass opacities and/or branching opacities. However, these findings are nonspecific and bear similarities to hypersensitivity pneumonia (HP), respiratory bronchiolitis, and/or respiratory tract infections [3]. Coupled with the fact that a significant majority of occupationally related patients have a long history of smoking, differential diagnosis has proven exceptionally challenging in past clinical practices. Additionally, while bronchoalveolar lavage fluid (BALF) ferritin analysis aids in differentiating between AWP and HP, its specificity remains limited due to the possibility of occupational sensitization triggering lymphocytosis [4]. Lung biopsy, though potentially insightful, faces limited acceptance owing to its invasive nature and potential complications. Hence, the quest for a novel imaging technique that can enhance the early identification of AWP holds paramount importance in improving diagnosis rates and avoiding unnecessary treatments.

Recent advancements in dual-energy CT (DECT) technology have transitioned CT from single-parameter to multi-parameter imaging. Single-source fast kVp-switching DECT enables material decomposition (MD) by acquiring additional projection data from a second tube voltage with photons of different X-ray energies. This technology allows for the quantification of components such as iodine, bone minerals, and blood [5–8]. We envision that the extraction and quantification of iron oxide in the lungs through basic material pairs could be more specific and therefore play a pivotal role in the early diagnosis of AWP, which has remained unexplored until now.

This study aims to evaluate the accuracy of quantifying the iron oxide deposition using Fe_2_O_3_-based MD images in DECT and its effectiveness in differentiating AWP from other conditions, and explore the correlation between lung iron oxide deposition and CT imaging characteristics in welders.

## Materials and methods

### In vitro experiments

Since the precise measurement of Fe_2_O_3_ concentration is crucial for the quantitative assessment of lesions, phantom experiments were first conducted to determine its accuracy. A set of 10 test tubes (30 mL) containing different known concentrations of Fe_2_O_3_ solutions (0 mg/mL, 1 mg/mL, 2 mg/mL, 3 mg/mL, 4 mg/mL, 5 mg/mL, 6 mg/mL, 8 mg/mL, 10 mg/mL, and 12 mg/mL) was scanned with the scanning range adjusted. Because Fe_2_O_3_ powder is not water-soluble, Fe_2_O_3_ particles settle at the bottom of the Fe_2_O_3_–water suspension within a few minutes; therefore, the tubes were scanned immediately after preparation. Additionally, triplicates were prepared for each solution to minimize preparation errors. Each tube was scanned three times to ensure reproducibility, using the same spectral imaging protocol with fast tube voltage switching between 80 kVp and 140 kVp and a collimation thickness of 0.625 mm. A radiologist with 5 years of experience used specialized software (GSI Viewer; GE HealthCare) to delineate the ROI for Fe_2_O_3_ concentrations and CT value measurements in each tube. The ROI was defined as a circle encompassing 80% of the cross-sectional area of the tube, excluding air bubbles and the tube wall (Fig. 2). This ROI was applied to two adjacent sections, and the average of the three measurements was used as the final value. To establish a correlation between the actual and measured Fe_2_O_3_ concentrations, a linear regression model was used.

### Participants

**Fig. 1:**
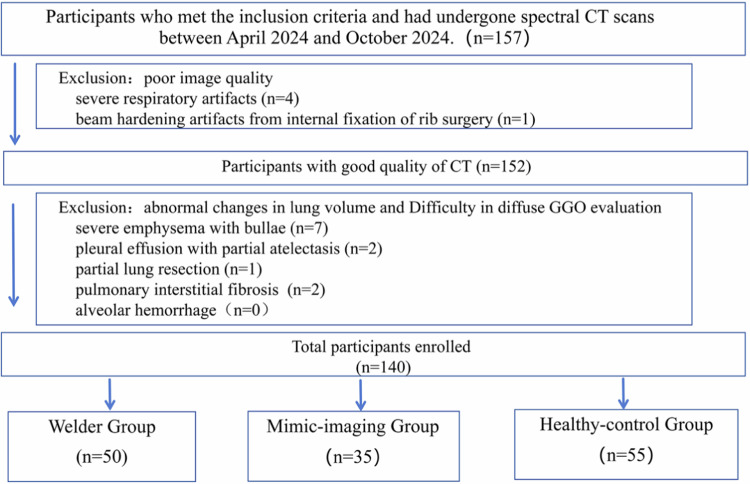
Flowchart of participant inclusions and exclusions

This prospective case-control study adhered to the Declaration of Helsinki 2013, received approval from our hospital’s ethics committee, and obtained written informed consent from all participants. The study was also registered with the Chinese Clinical Research Trial Center under registration number ChiCTR2400082410. Between April 2024 and October 2024, we recruited participants into three distinct groups: the arc-welder group, the mimic-imaging group, and the healthy control group. Participants in the arc-welder group were recruited in two ways. One was direct recruitment from local factories, and the other was to screen out people with a professional background in arc welding during the recruitment process of people with mimic-imaging characteristics. All participants had been welding continuously for three years or more. The mimic-imaging group included patients with diffuse lung lesions identified by chest CT scans one year prior to recruitment who volunteered to participate in the study. It also included hospitalized patients with diffuse lung lesions identified by DECT scans during the study period (with patient consent) who had no history of welding or other dust-related occupational diseases. The healthy control group recruited volunteers who had no history of occupational exposure to iron filings and no diffuse lesions on prior chest examination. Participants were excluded from the study based on the following criteria: (1) poor CT image quality (e.g., severe respiratory motion artifacts and severe metal artifacts); (2) presence of other pathologies that cause abnormal changes in lung volumes or interfere with the assessment of lung pathology (e.g., moderate to large pleural effusions, severe emphysema, interstitial fibrosis); and (3) surgical implants in the lung; (4) surgical implants in the lung; and (4) patients with radiological or clinical evidence of alveolar hemorrhage will be excluded. Patient recruitment is shown in Fig. 1. Patients’ age, gender, smoking history, and the presence of symptoms such as cough, chest tightness, and pain were collected.

### Imaging protocol

All participants underwent a non-enhanced CT scanning using a 256-row CT scanner (Revolution, GE HealthCare) in the gemstone spectral imaging (GSI) mode, with coverage from the thoracic inlet to the lung base. Participants were positioned supine with the head first. The scanning parameters included a pitch of 0.992:1, a tube current of 320 mA, and rapid tube voltage switching between 80 kVp and 140 kVp. Image reconstruction was performed using the adaptive statistical iterative reconstruction (ASIR) algorithm with a blending ratio of 60%. The slice thickness and interval for reconstruction were both set to 0.625 mm, and the standard reconstruction kernel was applied.

### Image analysis

#### Lung volumetry

All data were imported into a GE AW4.7 post-processing workstation (GE HealthCare). Images were reformatted using a lung window and displayed in the coronal plane with a slice thickness of 0.625 mm. Whole lung segmentation was performed on the monochromatic 70 keV images using a thresholding method with CT attenuation values ranging from −1023 HU to −170 HU (as per the device instructions).

#### Quantitative DECT parameters

The mean CT value and total lung volume (cm³) were measured on the monochromatic (Mono+) 70 keV images. The whole lung Fe_2_O_3_ density (*D*_Fe2O3_, mg/cm³), water density (*D*_water_, mg/cm³), and effective atomic number (*Z*_ef_) were measured using the GSI Volume viewer software. *D*_Fe2O3_ was measured on Fe_2_O_3_(water)-based paired basis material images, while *D*_water_ was measured on the water (Fe_2_O_3_)-based basis material images. The total Fe_2_O_3_ deposition mass of the whole lung (Total-Fe_2_O_3_) was calculated using: Total-Fe_2_O_3_ = *D*_Fe2O3_ × Lung volume. For the welder group, the largest solitary nodule visible on CT lung window images was selected for measuring its *D*_Fe2O3_.

#### Lesion diameter and imaging characteristics

Two radiologists, with 8 years and 13 years of chest diagnostic imaging experience, independently analyzed the lesion diameter and imaging characteristics of the welder and mimic-imaging groups. Images were graded based on the presence of diffuse lung lesions and the diameter of the majority of those lesions. Specifically, Grade 0 denoted the absence of diffuse lung lesions; Grade 1 indicated diffuse lesions with a diameter ≤ 3 mm (e.g., centrilobular ground-glass density micronodules or tiny tree-in-bud pattern); and Grade 2 represented diffuse lesions with a diameter > 3 mm (e.g., acinus ground-glass nodules or diffuse ground-glass opacities). Inter-observer agreement was statistically validated using weighted kappa coefficients. A standardised analysis protocol was implemented for lesions with subjective scores ≥ grade 1 in the welder group: Two principal investigators performed a joint review to identify the largest nodule. In case of disagreement, a third radiologist with more than 10 years of experience in thoracic imaging was invited to participate in the evaluation. For the quantitative measurement of GGO, thin-slice CT scans (slice thickness 0.625 mm) were used for multiplanar reconstruction (MPR), and the largest cross section in the transverse axis position was selected, and the transverse and anteroposterior diameters were measured, and the mean value was calculated as the diameter of the nodule. At the same time, using standardised lung window parameters (window width 1500 HU, window position −600 HU), a semi-automated tool was used at the workstation to delineate the ROI (region of interest) in the largest cross-section of the nodule and to obtain the *D*_Fe2O3_. Radiologists who participated in these evaluations were blinded to the clinical information and the results of the reference standard.

### Sample size calculation

Sample size was determined based on preliminary data using the PASS 2021 software for a one-way analysis of variance (ANOVA) with unequal variances (Welch’s test). With expected group *D*_Fe2O3_ means of 0.91, 0.43, and 0.44, and standard deviations of 0.43, 0.26, and 0.22 for the three groups, a total of 42 participants (14 per group) were needed to achieve 91% power at a significance level of 0.05.

### Statistical analysis

Statistical analysis was performed using SPSS V25.0 (IBM Corporation). The Shapiro–Wilk test was used to assess the normality of continuous variables. Variables that followed a normal distribution were analyzed using ANOVA, while those that were not normally distributed were examined with the Kruskal–Wallis test. The Chi-square test was used to analyze categorical variables such as gender and smoking history. The receiver operating characteristic (ROC) curves were plotted with MedCalc V22.023 to determine the optimal cut-off values for DECT parameters in separating welders and others. Spearman’s correlation coefficient assessed the correlations between subjective grade on lung lesions and whole lung *D*_Fe2O3_, Total-Fe_2_O_3_, and work duration, and the maximum orthogonal diameter of nodules and their *D*_Fe2O3_ values. *p* < 0.05 was considered statistically significant.

## Results

### In vitro experiments

**Fig. 2:**
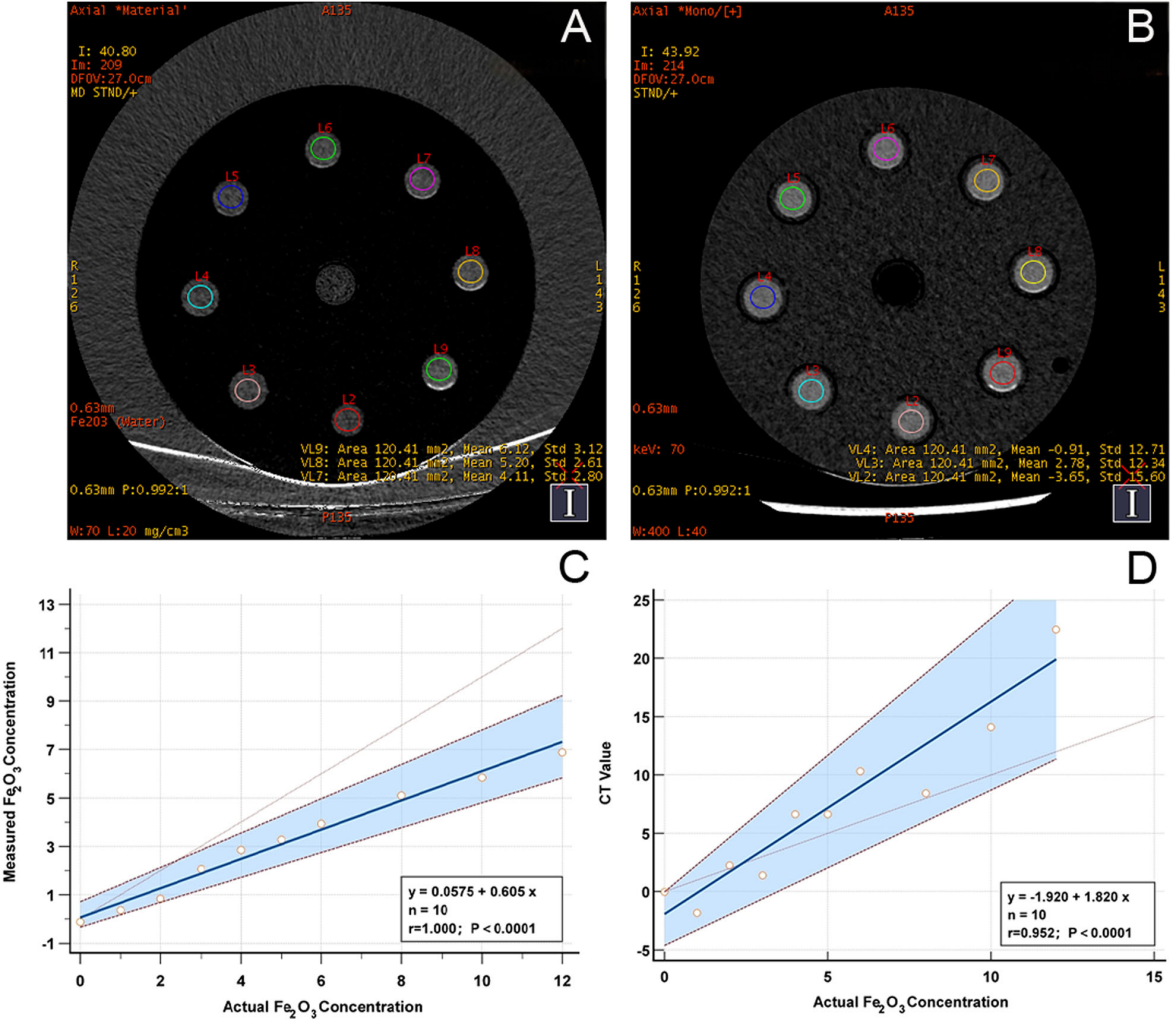
**A**, **B** Schematic representations of the actual measurements of Fe_2_O_3_ concentrations and CT values in an in vitro model, with regions of interest (ROIs) consistently located in the center of each test tube. Due to space limitations in the in vitro model, test tubes numbered 1 and 10 were additionally scanned and are not shown in the images. Although the solution was thoroughly shaken prior to scanning, water-insoluble Fe_2_O_3_ particles tended to settle as the concentration increased, particularly in tubes numbered 7–9. This sedimentation caused the measured Fe_2_O_3_ values to be closer to the actual values at lower concentrations, while the measured values fell below the actual values at higher concentrations. **C** The correlation between actual (*x*-axis) and measured (*y*-axis) Fe_2_O_3_ concentrations obtained from an in vitro analysis. **D** The relationship between actual Fe_2_O_3_ concentrations (*x*-axis) and CT values (*y*-axis) in the in vitro study

During the in vitro experiments, we observed a strong linear positive correlation between the measured Fe_2_O_3_ concentration and the actual Fe_2_O_3_ concentration, represented by the equation *y* = 0.0575 + 0.605*x*. The correlation coefficient, *r*, was found to be 1.00, with a *p*-value less than 0.01. The 70 keV CT values were also positively correlated with the actual Fe_2_O_3_ concentration, with an *r* of 0.952 (Fig. 2 and Table 1).

**Table 1:** Comparison of actual and measured Fe_2_O_3_ concentrations and CT values in in vitro DECT experiments

Sample no.	Actual Fe_2_O_3_ concentration (mg/cm^3^)	Measured Fe_2_O_3_ noncentration (mg/cm^3^)	CT value (HU)
1	0	−0.13 ± 0.27	−0.02 ± 2.24
2	1	0.35 ± 0.05	−1.82 ± 1.55
3	2	0.83 ± 0.16	2.26 ± 5.04
4	3	2.06 ± 0.27	1.38 ± 2.32
5	4	2.85 ± 0.21	6.65 ± 2.80
6	5	3.28 ± 0.38	6.64 ± 2.10
7	6	3.93 ± 0.19	10.34 ± 2.60
8	8	5.09 ± 0.33	8.44 ± 2.51
9	10	5.85 ± 0.23	14.12 ± 2.35
10	12	6.88 ± 0.36	22.46 ± 2.47

### Participants

As shown in Fig. 1, a total of 140 participants were included in the analysis, exceeding the sample size determined by preliminary calculations. The study comprised 50 participants in the welder group, 35 in the mimic-imaging control group, and 55 in the healthy control group. Statistically significant differences were observed between the groups in terms of gender and age (*p* = 0.002; *p* = 0.003), while no significant differences were found regarding smoking status (all *p* > 0.05).

### Quantitative DECT parameters

**Fig. 3:**
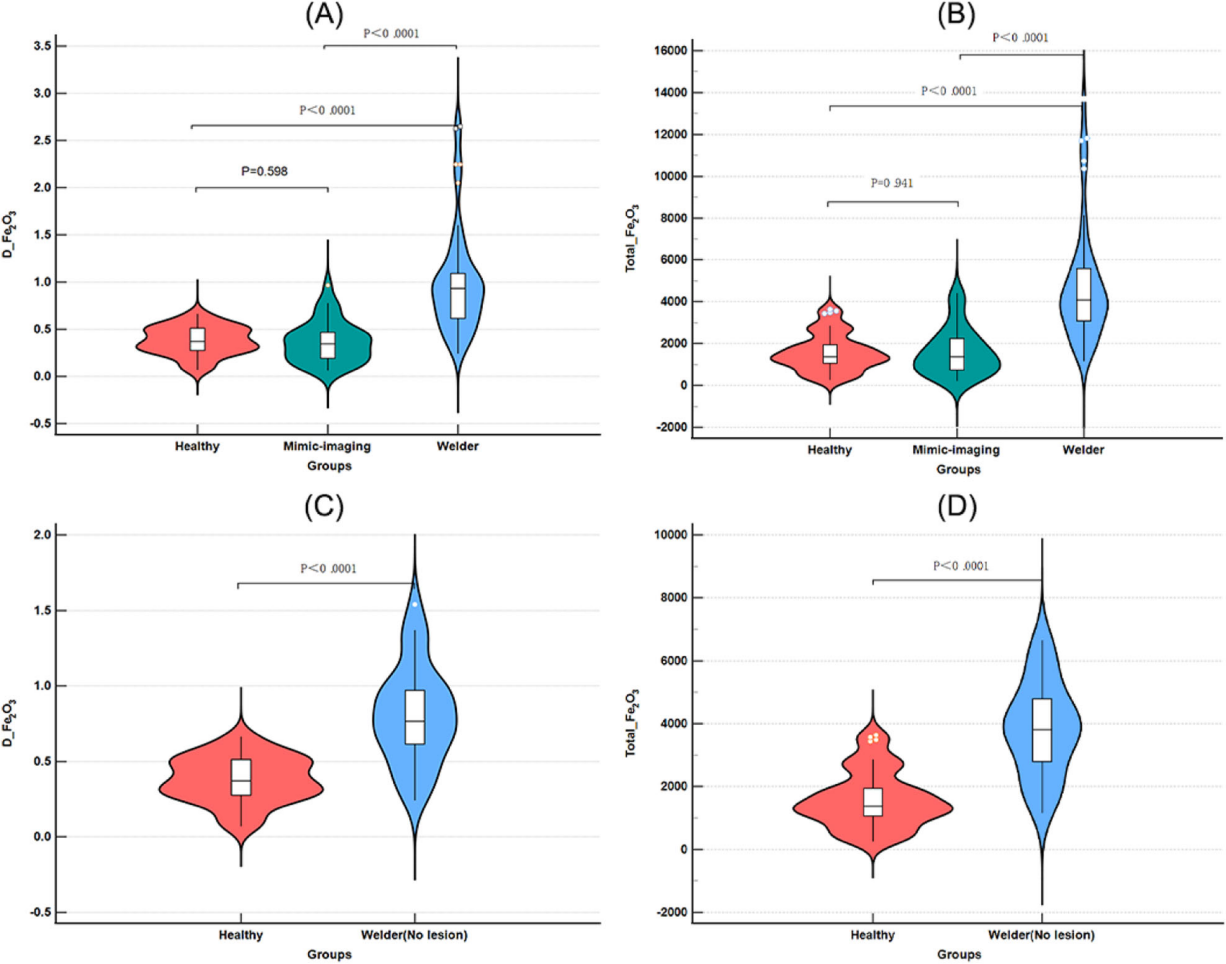
Comparison of *D*_Fe2O3_ (**A**) and Total Fe_2_O_3_ (**B**) content across the three groups. **C**, **D** The comparison of *D*_Fe2O3_ and total Fe_2_O_3_ content in the welder group without lesions and the healthy control group

Compared to the mimic-imaging group and the healthy control group, the welder group exhibited significantly elevated whole lung *D*_Fe2O3_ and Total-Fe_2_O_3_, with values of (welder: 0.934 ± 0.50 mg/cm³) compared to (mimic-imaging: 0.346 ± 0.28 mg/cm³) and (healthy: 0.371 ± 0.24 mg/cm³) for *D*_Fe2O3_, and (welder: 4082.62 ± 2503.13 mg) compared to (mimic-imaging: 1376.14 ± 1514.93 mg) and (healthy: 1374.14 ± 896.20 mg) for Total-Fe_2_O_3_ (all *p* < 0.001). In addition, a comparison of the Total-Fe_2_O_3_ and *D*_Fe2O3_ content between the welder group without lesions and the healthy control group also revealed statistically significant differences (*p* < 0.01). The lung volume exhibited slight statistical differences among the three groups, whereas no statistical difference was observed in the 70 keV-CT values, *D*_water_, and *Z*_ef_ among the three groups (Fig. 3 and Table 2). To evaluate the independent impact of smoking behavior on pulmonary iron metabolism, we conducted smoking subgroup analyses, including: Welders who smoke vs non-smoking welders, Smoking controls vs non-smoking controls, and non-smoking welders vs smoking mimic controls. The results demonstrated that no statistically significant associations were observed between smoking status and key iron deposition parameters (*D*_Fe2O3_ and Total-Fe_2_O_3_) in the welder group or either control subgroup (all comparisons *p* > 0.05). However, a significant difference (*p* < 0.01) was identified between non-smoking welders and smoking mimic controls (Fig. S1).

**Table 2:** Participant characteristics, stratified by iron dust-contacting and imaging features

Characteristic	Welder group (*n* = 50)	Mimic-imaging group (*n* = 35)	Healthy-control group (*n* = 55)	*p*-value
Gender (M:F)	47:3^c^	34:1^c^	41:14^ab^	0.002
Age (y)^†^	44.5 (20.25)^b^	51.00 (10.00)^a^	48.00 (19.00)	0.003
Presence of smoking	31/50	27/35	37/55	0.34
Spectral parameters				
* D*_Fe2O3_ (mg/cm^3^)^†^	0.934 (0.50)^b,c^	0.346 (0.28)^a^	0.371 (0.24)^a^	< 0.01
70 keV-CT (HU)^†^	−817.50 (45.75)	−817.00 (42.00)	−818.00 (57.00)	0.49
* D*_water_^†^ (mg/cm^3^)^†^	183.85 (43.90)	182.00 (52.10)	181.60 (56.90)	0.62
Lung volume (cm^3^)^†^	4851.71 (1447.37)^c^	4607.41 (1875.92)	4105.88 (1790.84)^a^	0.02
* Z*_ef_^†^	7.50 (0.34)	7.35 (0.30)	7.36 (0.24)	0.32
Total-Fe_2_O_3_ (mg)^†^	4082.62 (2503.13)^b,c^	1376.14 (1514.93)^a^	1374.14 (896.20)^a^	< 0.01
Subjective grading on imaging features				
Grade 0 (no lesion)	28/50	0/35	55/55	
Grade 1 (mostly *d* ≤ 3 mm)	16/50	28/35	0/55	
Grade 2 (mostly d > 3 mm)	6/50	7/35	0/55	
Presence of symptoms	24/50	12/35	0/55	

Unless otherwise specified, data are the number of participants

^†^ Parameters are expressed as median (IQR)

^a^ Statistical difference (*p* < 0.05) compared with the welder group

^b^ Statistical difference (*p* < 0.05) compared with the mimic imaging group

^c^ Statistical difference (*p* < 0.05) compared with the healthy control group

### Diagnostic performances of DECT quantifications

**Fig. 4:**
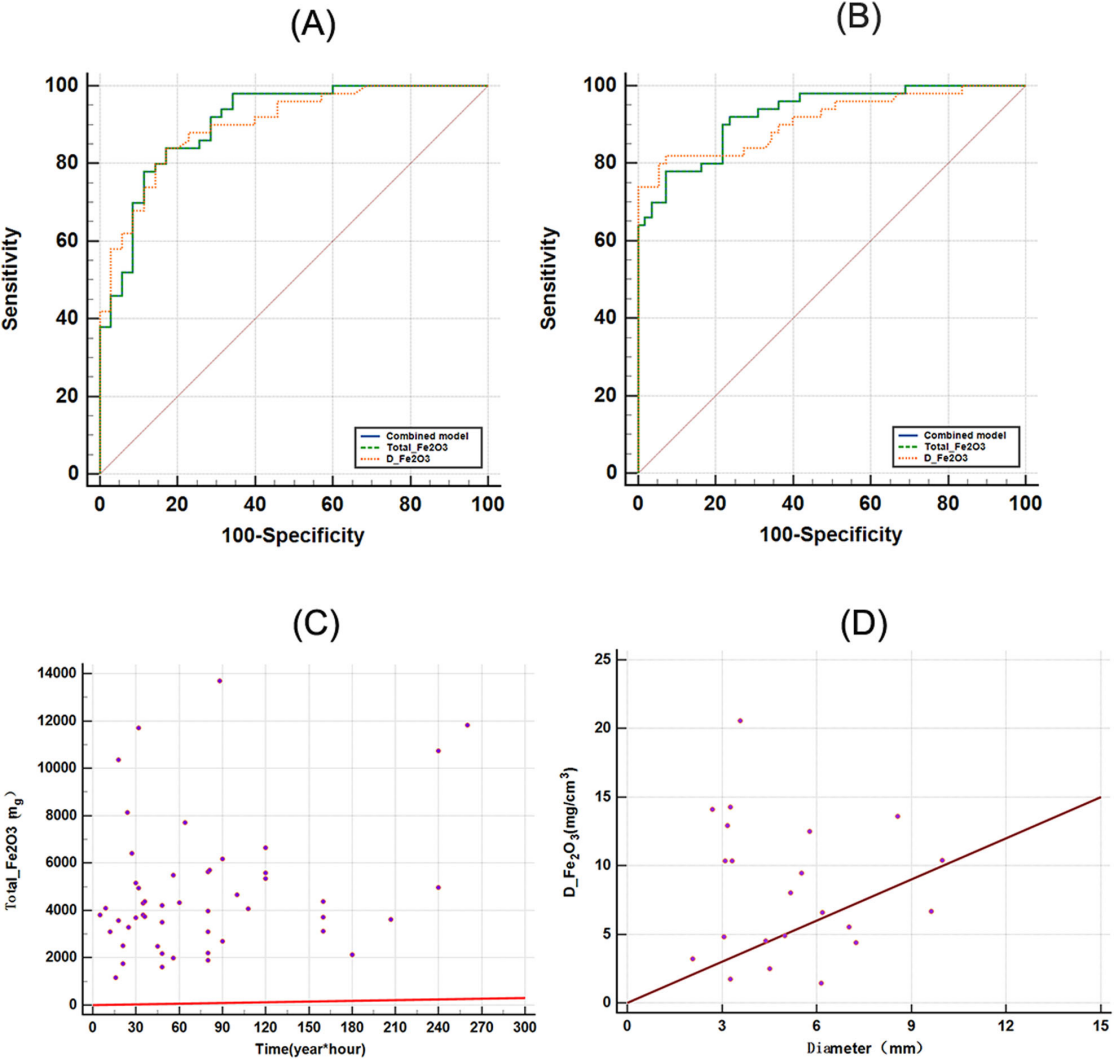
**A** ROC curve of *D*_Fe2O3_ and Total-Fe_2_O_3_ in differentiating mimic-imaging vs welder group. **B** ROC curves of *D*_Fe2O3_ and Total-Fe_2_O_3_ in distinguishing the healthy and welder groups. **C** Scatter plot illustrating the correlation between whole lung Total-Fe_2_O_3_ and work duration. **D** Scatter plot showing the maximum diameter of the largest lung nodule and *D*_Fe2O3_ content within the nodule

ROC curves were generated using *D*_Fe2O3_ and Total-Fe_2_O_3_ in differentiating the healthy vs welder and mimic-imaging vs welder groups. The AUCs with Total-Fe_2_O_3_ were higher than those with *D*_Fe2O3_, with values of 0.927 [95% CI: 0.859, 0.969] and 0.906 [95% CI: 0.823, 0.958], respectively. The optimal cut-off value for differentiating welders from mimic-imaging controls using Total-Fe_2_O_3_ was 2411.49 mg, leading to 84% in sensitivity and 82.86% in specificity, while that for welders from healthy controls was 2863.31 mg, resulting in 78% in sensitivity and 92.73% in specificity. The cutoff values for *D*_Fe2O3_ were determined to be 0.565 mg/cm³ (*p* < 0.01) for the healthy vs welder group, yielding a sensitivity of 82.00% and specificity of 92.73%, and 0.488 mg/cm³ (*p* < 0.01) for the mimic-imaging vs welder group, resulting in a sensitivity of 84.00% and specificity of 82.86%. Figure 4 and Table 3 present the detailed ROC analysis results for using *D*_Fe2O3_ and Total-Fe_2_O_3_.

**Table 3:** ROCs analysis in differentiating welders from other conditions

	Model	AUC	Cutoff	Sensitivity	Specificity	*p*
Mimic-imaging vs welder group	*D*_Fe2O3_	0.900 [95% CI: 0.816, 0.954]	0.488 mg/cm³	84.00%	82.86%	< 0.01
	Total-Fe_2_O_3_	0.906 [95% CI: 0.823, 0.958]	2411.49 mg	84.00%	82.86%	< 0.01
Healthy vs welder group	*D*_Fe2O3_	0.911 [95% CI: 0.840, 0.958]	0.565 mg/cm³	82.00%	92.73%	< 0.01
	Total-Fe_2_O_3_	0.927 [95% CI: 0.859, 0.969]	2863.31 mg	78.00%	92.73%	< 0.01

### Correlations between imaging features, work duration time, and DECT quantifications

The inter-observer agreement for subjective lesion grading demonstrated consistency with a weighted kappa of 0.81 (95% CI: 0.71–0.92). The Spearman’s correlation coefficient (Rs) analysis revealed a coefficient of 0.357 (*p* = 0.011) between subjective imaging grade on lung lesion and *D*_Fe2O3_ in the welder group, indicating a weak but statistically significant correlation. Moreover, the analysis showed no significant correlation (rho = −0.075, *p* = 0.74) between the maximum orthogonal diameter of the largest nodule and its *D*_Fe2O3_. Additionally, the association between Total-Fe_2_O_3_ and work duration time in the welder group was also not statistically significant (rho = 0.262, *p* = 0.067) (Fig. 4).

## Discussion

Currently, there is no single test that serves as the gold standard for diagnosing AWP. The diagnosis relies on a combined evaluation of clinical history, imaging examination, and pathology. The results of our study showed that the values of the whole lung *D*_Fe2O3_ and Total-Fe_2_O_3_ on the iron oxide-based MD images in DECT were significantly higher in the welder group compared with other control groups (*p* < 0.01). In addition, the AUC values for distinguishing welders from others with both *D*_Fe2O3_ and Total-Fe_2_O_3_ were quite high, exceeding 0.9. In particular, the AUC value of using Total-Fe_2_O_3_ was slightly higher than using *D*_Fe2O3_. When differentiating between the welder occupational group and the analogue imaging group, both Total-Fe_2_O_3_ and *D*_Fe2O3_ achieved a sensitivity of 84.00% and a specificity of 82.86%. All these aspects highlighted the potentially excellent ability of *D*_Fe2O3_ and Total-Fe_2_O_3_ to differentiate AWP from other diseases with similar imaging manifestations. However, the sensitivity of using Total-Fe_2_O_3_ was slightly lower than using *D*_Fe2O3_ when specifically discriminating between healthy subjects and welders. Considering that *D*_Fe2O3_ is more convenient to use, we advocate the measurement of *D*_Fe2O3_ for the differential diagnosis of AWP. In addition, we recommend that occupational health screening of welders include measurement of *D*_Fe2O3_ and Total-Fe_2_O_3_, and that variations in lung iron content be closely monitored, particularly in individuals with lesion-negative imaging characteristics. This recommendation is supported by the finding that the comparison of Total-Fe_2_O_3_, and *D*_Fe2O3_ levels between the lesion-free welder group and the healthy control group revealed statistically significant differences (*p* < 0.01). In addition, *D*_Fe2O3_ and Total-Fe_2_O_3_ showed a remarkable specificity of 92.73% in discriminating between the healthy and welder groups.

**Fig. 5:**
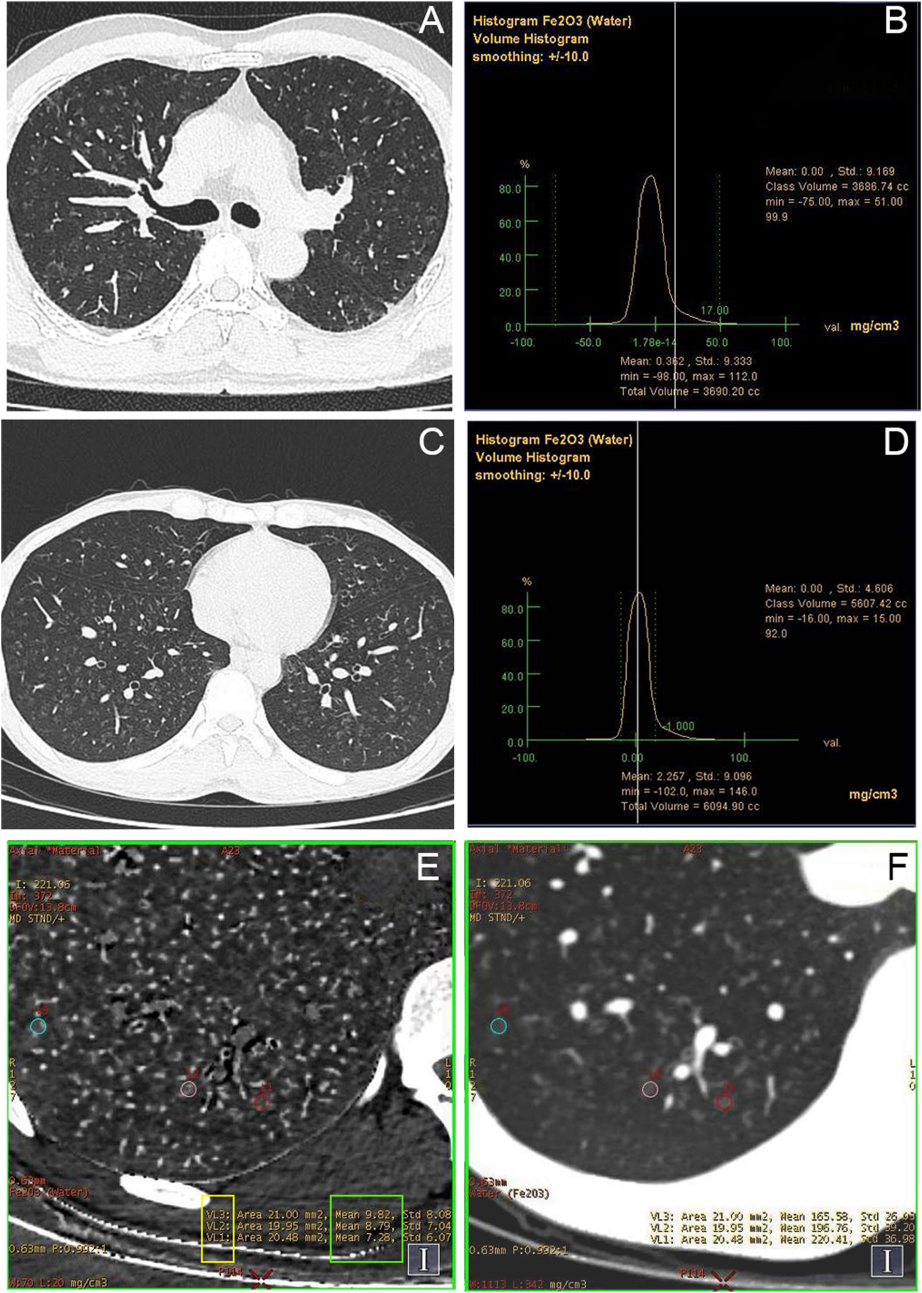
A 45-year-old male patient underwent a chest CT scan, which revealed diffuse centrilobular ground-glass density micronodules in both lungs (**A**). The patient had no history of occupational exposure to welding fumes or other dust-related diseases. Respiratory bronchiolitis was suspected due to the patient’s 20-year history of smoking, with a total lung *D*_Fe2O3_ of 0.362 mg/cm^3^, which is below the cutoff value (**B**). A 39-year-old male, with an 8-year history of welding, presented with vague chest pain for several years and a sudden onset of burning chest pain lasting more than 1 day. The chest CT shows the same findings as in the A-view patient (**C**), which did not change significantly after several diagnostic and therapeutic interventions. The total lung *D*_Fe2O3_ was 2.257 mg/cm^3^, which is significantly higher than the cutoff (**D**). The *D*_Fe2O3_ in the ROI lesion (**E, F**) was higher than that in the total lung *D*_Fe2O3_ (**D**). This further supports the diagnosis of AWP

**Fig. 6:**
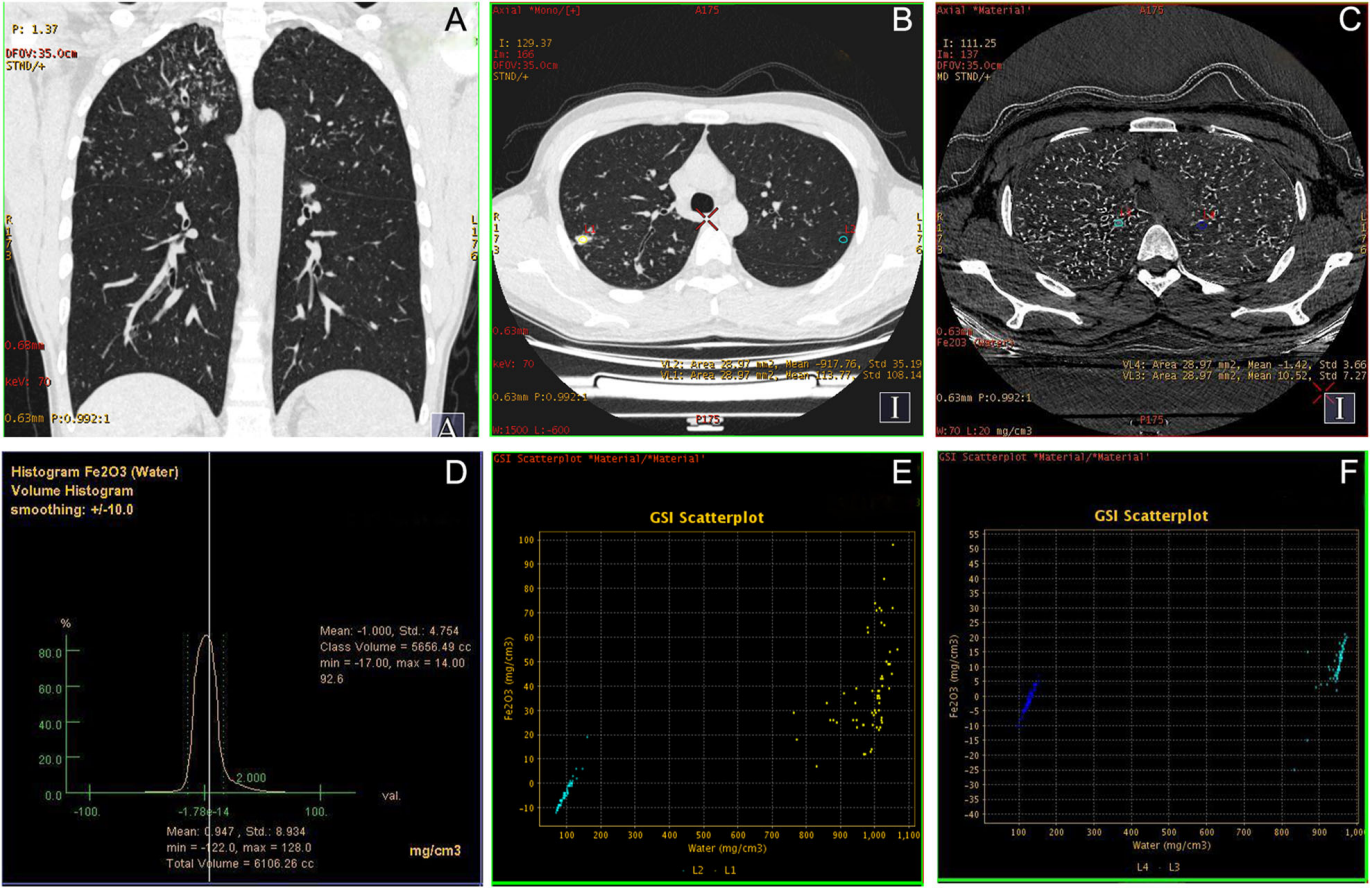
A 28-year-old male with a 9-year occupational history as a welder was diagnosed with tuberculosis six months ago, following the appearance of multiple tree-in-bud pattern lesions in both upper lungs on chest CT (**A**). However, the lesions showed no improvement despite long-term anti-tuberculosis treatment. The GSI scatter plot reveals that the *D*_Fe2O3_ in the lesion is significantly higher than that in the contralateral side (**B**, **C**, **E**, **F**). The *D*_Fe2O3_ in the entire lung was 0.947 mg/cm^3^ (**D**). These findings suggest that the lung lesions may represent iron deposition rather than tuberculosis

AWP patients often exhibit no symptoms or only mild ones during the early stages [9, 10]. Thus, it is challenging to diagnose AWP solely based on clinical symptoms and conventional imaging techniques. This study revealed that welders suspected of having AWP exhibited symptoms similar to those observed in patients with various lung diseases. These symptoms included nonspecific asymptomatic cases (26/50, 52%), as well as cough, chest tightness, and pain (24/50, 48%), among others. In the past, research on AWP primarily focused on traditional image feature analysis. Akira et al assessed thin-section CT findings of 21 arc welders and found that the most common findings were ill-defined micronodules diffusely distributed in the lungs (*n* = 15, 71.4%). Some of the micronodules appeared as fine branching structures and tended to show a centrilobular distribution [11]. Zhuang et al evaluated CT findings among electric welders and observed that the most prevalent manifestations were centrilobular ground-glass density micronodules (*n* = 44, 42.7%) and the tiny tree-in-bud pattern (*n* = 63, 61.1%). Comparatively, acinus ground-glass nodules (*n* = 7, 6.8%), diffuse ground-glass opacities (*n* = 10, 9.7%), and dense micronodules (*n* = 6, 5.8%) were relatively infrequent [9]. These findings are consistent with our results. However, these observations are also commonly seen in smoking-induced lung changes, such as respiratory bronchiolitis and desquamative interstitial pneumonia (DIP). The pathological basis is similar to that of AWP, involving the accumulation of macrophages within the lumens of distal airways and peribronchiolar airspaces [12]. The term “smoker’s macrophages” in DIP/RB-ILD pathology describes pigmented airspace macrophages characterized by pale eosinophilic cytoplasm with abundant yellowish-brown to light brown granules [13–15]. Although these pigments show Prussian blue positivity (posing diagnostic challenges in distinguishing them from hemosiderin), they are not hemosiderin [12]. Our findings—no significant links between smoking status and iron deposition parameters ($D_{{{\rm{Fe}}}_2{{\rm{O}}}_3}$ and Total-Fe_2_O_3_)—further corroborate this conclusion. By quantifying iron content in the lung, our study has the capability to differentiate AWP from other comparable imaging conditions (Fig. 5), thus addressing a long-standing issue that has perplexed radiologists. For patients exhibiting atypical AWP imaging findings, early diagnosis can be notably enhanced by assessing *D*_Fe2O3_ levels in the affected area, thereby preventing unnecessary overtreatment (Fig. 6).

Zhao et al established, through bronchofiberscope biopsy of lung areas affected by welder’s pneumoconiosis, that dust spots are the primary pathological basis for centrilobular ground-glass density micronodules and the tiny tree-in-bud pattern [16]. Furthermore, acinar ground-glass nodules and diffuse ground-glass opacities may be associated with reactive alveolitis induced by short-term exposure to high concentrations of welding fumes [16]. These pathological foundations clarify why our results show no correlation between the maximum orthogonal diameter of the nodule and its *D*_Fe2O3_ concentration, whereas a weak correlation was observed between lesion grade and *D*_Fe2O3_ specifically, the size of centrilobular lesions does not reflect the extent of iron dust spots, possibly due to an alveolar inflammatory response.

In this study, in vitro experiments played a critical role in validating the use of chest DECT for the extraction of iron substances in the diagnosis of AWP. We observed a strong linear correlation between the measured and actual concentrations of Fe_2_O_3_, with the correlation equation being *y* = 0.0575 + 0.605*x*, a correlation coefficient (*r*) of 1.00, and a *p*-value of less than 0.01. Despite thoroughly shaking the solution before scanning, water-insoluble Fe_2_O_3_ particles tended to settle with increasing concentration. This settling effect caused the measured Fe_2_O_3_ concentration to be closer to the actual value at lower concentrations, while at higher concentrations, the measured value was lower than the actual concentration. It is important to note that such settling does not occur with iron deposition in the lungs, which suggests that this effect will not impact the clinical diagnosis. Additionally, CT values were measured and found to show a positive correlation with the actual Fe_2_O_3_ concentration. However, it is important to note that CT values reflect the overall density of all substances in the sample, not just the concentration of Fe_2_O_3_. In subsequent comparisons of whole-lung 70 keV-CT values across the three groups, no statistically significant differences were observed, further confirming that CT values should be considered reference indicators rather than standalone measures of actual Fe_2_O_3_ concentration. Moreover, the in vitro experiments also helped calibrate the imaging parameters and improved the precision of Fe_2_O_3_ quantification. This ensured the effectiveness and accuracy of the imaging technique, thereby reducing potential errors in the interpretation process. Thus, it is recommended to perform in vitro experiments with Fe_2_O_3_ standard samples of different concentrations for calibration after the alterations of scanning parameters, or the changes of device components such as the CT tube, detector, and collimator etc.

This study has several limitations. First, during the whole-lung segmentation process, blood vessels could not be excluded, leading to the inclusion of Fe_2_O_3_ content from the blood. As a result, the *D*_Fe2O3_ and Total-Fe_2_O_3_ measurements may not fully reflect the true iron content in the lungs. However, this methodology was consistently applied to both the study and control groups, and the relative errors introduced should be minimal. Second, as a prospective study, obtaining pathological results was challenging. More than half of the welders recruited did not show imaging abnormalities. Among those who did, clinical symptoms were mild, and many patients declined biopsy due to concerns about potential complications. This highlights the need for non-invasive diagnostic methods for AWP, which further emphasizes the importance of our research. While pathological examination is an essential diagnostic tool, it is not the sole gold standard for AWP but rather one of several supportive diagnostic modalities. To address this limitation, we employed in vitro experiments to validate the imaging techniques and assess their reliability. Third, due to the diffuse nature of the lesions, accurate measurement of *D*_Fe2O3_ and the nodule diameter was challenging. Fourth, there is a potential for recall bias in the collection of clinical data, especially regarding self-reported working hours. Lastly, certain rare forms of alveolar hemorrhage may exhibit imaging overlap with AWP and similarly increase pulmonary iron content. Due to their rarity, these conditions were not included in our differential diagnosis. The potential diagnostic value of iron quantification in distinguishing such cases warrants further investigation in future large-scale studies.

In conclusion, the quantification of pulmonary *D*_Fe2O3_ and Total-Fe_2_O_3_, and the use of DECT scans may aid in the differential diagnosis of AWP. The size of centrilobular lesions may not be related to iron dust deposition.

## Supplementary information

ELECTRONIC SUPPLEMENTARY MATERIAL

## Abbreviations

ANOVA: Analysis of variance
AWP: Arc-welders’ pneumoconiosis
DECT: Dual-energy CT
*D*_Fe2O3_: Whole-lung Fe_2_O_3_ density
HP: Hypersensitivity pneumonia
MD: Material decomposition
ROC: Receiver operating characteristic
Total-Fe_2_O_3_: Total Fe_2_O_3_ mass
