# Supplementary material for: Quantifying whole lung iron oxide deposition with dual-energy CT for diagnosis of arc-welders’ pneumoconiosis
Source: Eur Radiol. 2025 Jul 25;36(1):98–110. doi: 10.1007/s00330-025-11839-z (PMC12712031; doi:10.1007/s00330-025-11839-z)
Supplement: Supplementary file 1 — ELECTRONIC SUPPLEMENTARY MATERIAL [file 330_2025_11839_MOESM1_ESM.pdf]

Quantifying Whole Lung Iron Oxide Deposition with Dual-Energy CT for Diagnosis of Arc-Welders' Pneumoconiosis

ELECTRONIC SUPPLEMENTARY MATERIAL

Figure S1. Distribution Patterns of $D_{Fe_2O_3}$ and Total-Fe_2O_3 in Smoking Subgroups

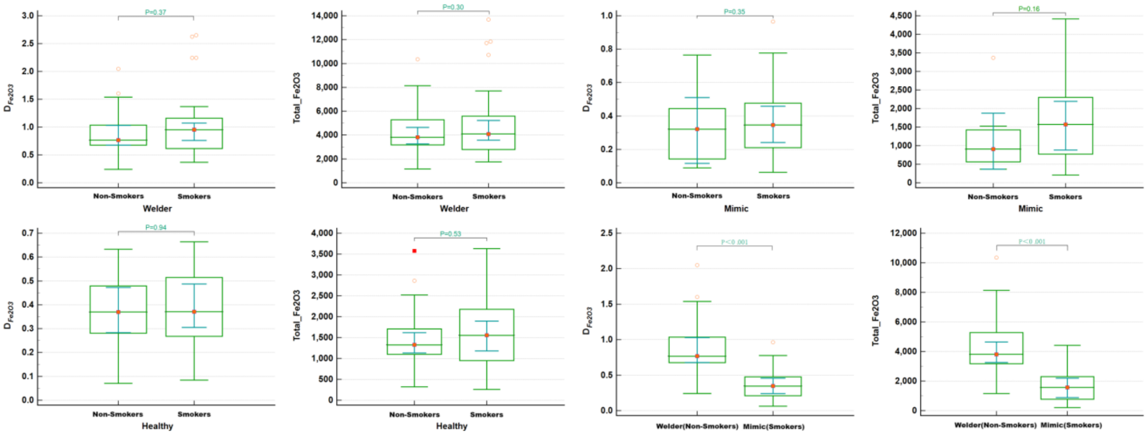

Eur Radiol (2025) Wang W, Ou Y, Lu L, et al.
